# Exploiting the antiviral potential of intermetallic nanoparticles

**DOI:** 10.1007/s42247-021-00306-2

**Published:** 2021-11-09

**Authors:** Rupy Kaur Matharu, Yuen-Ki Cheong, Guogang Ren, Mohan Edirisinghe, Lena Ciric

**Affiliations:** 1 Department of Mechanical Engineering, University College London, Torrington Place, London, WC1E 7JE UK; 2 Department of Civil, Environmental and Geomatic Engineering, University College London, Gower Street, London, WC1E 6BT UK; 3 School of Engineering and Computer Science, University of Hertfordshire, Hatfield, AL10 9AB UK

**Keywords:** Nanoparticles, Antiviral, Intermetallic, Alloys, Silver, Copper, Zinc, Composites

## Abstract

Viral pandemic outbreaks cause a significant burden on global health as well as healthcare expenditure. The use of antiviral agents not only reduces the spread of viral pathogens but also diminishes the likelihood of them causing infection. The antiviral properties of novel copper-silver and copper-zinc intermetallic nanoparticles against *Escherichia coli* bacteriophage MS2 (RNA virus) and *Escherichia coli* bacteriophage T4 (DNA virus) are presented. The intermetallic nanoparticles were spherical in shape and were between 90 and 120 nm. Antiviral activity was assessed at concentrations ranging from 0.05 to 2.0 wt/v% for 3 and 24 h using DNA and RNA virus model organisms. Both types of nanoparticles demonstrated strong potency towards RNA viruses (> 89% viral reduction), whilst copper-silver nanoparticles were slightly more toxic towards DNA viruses when compared to copper-zinc nanoparticles. Both nanoparticles were then incorporated into polymeric fibres (carrier) to investigate their antiviral effectiveness when composited into polymeric matrices. Fibres containing copper-silver nanoparticles exhibited favourable antiviral properties, with a viral reduction of 75% after 3 h of exposure. The excellent antiviral properties of the intermetallic nanoparticles reported in this study against both types of viruses together with their unique material properties can make them significant alternatives to conventional antiviral therapies and decontamination agents.

## Introduction

Viruses are pathogenic microorganisms that are made up of a core genetic material, either DNA or RNA, surrounded by a protective protein coat known as a capsid. They cause disease by diffusion, penetrating and reproducing inside host cells, consequently causing biological damages. Viral pathogens are the most abundant life form and infect virtually all organisms [1]. It has been estimated that there are 10^31^–10^32^ virus particles in the biosphere [2, 3]. This number surpasses the number of host cells at least by one order of magnitude. As a result, nearly every living organism is under constant attack, so much so, viruses are responsible for one of the greatest evolutionary pressure on cellular entities.

Though the creation of vaccines has led to the eradication and reduction of many viral infections, such as smallpox and measles, there are a large number of viral pathogens with no available treatment, including adenovirus, coronavirus, herpes simplex virus, Marburg virus and norovirus. These pathogens are easily spread from one person to another, through direct and indirect exposure.

Adenovirus is a non-enveloped double-stranded DNA virus that is associated with a wide range of human illnesses, including respiratory, ocular, urinary tract and gastrointestinal diseases. Adenovirus is icosahedral in shape and has a diameter between 90 and 100 nm. It has been detected in water supplies throughout the world [4–10]. Outbreaks typically occur in childcare centres, including day cares and schools, healthcare institutions and military settings [11]. Adenovirus outbreaks from recreational waters, such as swimming pools, are more frequent than any other waterborne virus [11–13]. In addition, two drinking water outbreaks have been documented for adenovirus in Europe [5, 14]. A study of intestinal disease in the UK identified adenovirus as the second most common cause of viral gastroenteritis in children [15]. Adenovirus is a robust virus that can survive well in the environment. In fact, it is more common, by tenfold, in sewage, sewage-contaminated and surface waters than enteroviruses.

Coronaviruses, a group of enveloped RNA viruses belonging to the Coronaviridae family, are a well-established etiological agent of respiratory diseases. They have a large pleomorphic spherical structure with bulbous surface projections and an average diameter between 80 and 120 nm [16]. Whilst there have been reported cases of adult infection worldwide, coronavirus predominantly strikes in elderly, people with underlying diseases and immunocompromised populations. There have been five major outbreaks caused by three strains over the last 20 years. The first outbreak of severe acute respiratory syndrome (SARS-CoV) was recorded in November 2002 and infected over 8000 people, approximately 10% of whom died [17, 18]. The largest outbreak (SARS-CoV-2) occurred in December 2019, causing 15,716,043 laboratory-confirmed infections and killing approximately 637,618 people (fatality rate of 4.1%) after 6 months [19, 20].

With no available treatment for the aforementioned infectious diseases and many other viruses, outbreaks caused by pathogenic viruses continue to emerge. Many researchers and pharmaceutical companies are seeking new antiviral agents, as they are considered an important adjunct to vaccination to reduce the medical and economic burden of viral infection. The use of antivirals not only reduces the spread of these viral pathogens but also diminishes the likelihood of them causing infections. Nanomaterials have shown remarkable potential for tackling various aspects of infections [21–25]. They have been demonstrated to possess inherent antimicrobial properties that are not or rarely expressed in their bulk form within the given timeframe. In particular, intermetallic materials are a type of metallic alloy that is composed of two or more elemental metals. Numerous studies have demonstrated that metals, particularly copper, bear strong antimicrobial properties. As a result, the interest in developing copper-based alloys has been increasing over the last few years. The virucidal properties of copper nanoparticles are thought to be due to (i) the degradation of viral proteins and (ii) the generation of hydroxyl radicals [22]. The major antiviral mechanisms of silver and zinc nanoparticles are likely the physical inhibition of binding between the virus and the host cell [22]. In this research, for the first time, the antiviral properties of copper-silver and copper-zinc alloy composites were studied, as both silver and zinc have also demonstrated promising antimicrobial activity. Existing literature only explores the antiviral properties of single elemental nanoparticles, whereas here, the antiviral properties of intermetallic nanoparticles are presented. The intermetallic nanoparticles were then incorporated into polymeric fibres, serving as a handleable carrier, to determine if they remain effective, thus demonstrating their usability. Antiviral studies were carried out using bacteriophages. Bacteriophages are viruses that are infectious to bacteria cells and are therefore suitable model microorganisms for virus research. They are safe to use and display structural features that are similar to human and animal viruses.

## Materials and methods

### Nanoparticles and their dispersion

Intermetallic copper-silver and copper-zinc nanoparticles were obtained from Sigma-Aldrich (Dorset, UK). For powder characterisation, nanoparticles were used as received, unless otherwise indicated. For antiviral tests, each sample was prepared in situ by suspending 0.1% wt/v (1000 ppm) of nanopowder into pure water (Acros, Thermo Fisher Scientific Brand, Loughborough, UK), which was subsequently dispersed using a liquid processor (Sonics & Materials®, CT, USA) performing at a 55% working power and a pulse sequence (20 s ON, 5 s OFF) for 2 min 20 s.

#### Scanning electron microscopy

The morphology of intermetallic copper-silver and copper-zinc nanoparticles was assessed using scanning electron microscopy (SEM). Nanopowder (2 mg) was secured onto a carbon-based adhesive substrate and positioned on a specimen stage; both samples were subjected to alternate vacuum-argon environment prior to sputter-coating with 20-nm nanogold using an Emitech SC7620 coater (Quorum Technologies, Ltd., East Sussex, UK). All SEM analyses were acquired using a JEOL JCM-5700 (JEOL Ltd., Welwyn Garden City, UK) instrument and images were collected using the built-in software with an accelerating voltage of 20 kV. Adobe® Lightroom CC (Adobe Inc., CA, USA) was used to improve contrast and quality of images.

#### Fourier-transform infrared and raman spectroscopy

Fourier-transform infrared (FTIR) spectra of intermetallic copper-silver and copper-zinc nanoparticles were acquired using a PerkinElmer Frontier FT-IR/FIR spectrometer (Coventry, UK) equipped with an attenuated total reflectance (ATR) accessory. Powder samples were loaded directly onto the diamond crystal stage and secured by a compressor rod. Blanks were assessed prior to measurement of each analyte; all data were acquired using built-in software ‘IRWinLab’ and 64 scans were collected. All spectra were measured at a wavenumber range between 3500 and 500 cm^−1^ and further processed using OrignLab© software (OriginLab©, MA, USA).

Raman spectra were obtained by a Renishaw inVia Raman microscope (Gloucestershire, UK) with built-in WiRE 3.4 software. All measurements were performed at 785-nm excitation wavelength and a 2-mW power laser. All powder samples were loaded on a microscope slide with an approximate examined area of 20 × 20 μm^2^ and each measurement was taken after an average of 20 scans. The data were further processed using the OriginLab® program (OriginLab©, MA, USA) and all visible Raman shifts were studied against references available in the BioRed® database (PA, USA).

#### Inductive coupled plasma-optical emission spectroscopy

To prepare samples for elemental and metal trace analysis, copper-silver  and copper-zinc nanoparticles (50 mg) were each mixed with 5 mL of high-purity nitric acid (99.999% trace metal basis, Sigma-Aldrich, Dorset, UK) and then subjected to CEM MARS Xpress microwaves for 55 min to obtain fully digested homogeneous solutions. The samples were then diluted × 10 with 2% nitric acid and filtered using 0.1-µm syringe filter (Merck Millipore, Hertfordshire, UK) prior to analysis. Emission intensities of each wavelength from all samples were measured and were acquired in triplicate. To obtain calibration curves of copper, silver and zinc, inductive coupled plasma (ICP) analytical stock solutions (1000 ppm in 2% HNO_3_) of ‘copper’, ‘silver’ and ‘zinc’ (Sigma-Aldrich, Dorset, UK) were used to prepare calibration standards at concentrations 10, 5, 1, 0.5, 0.1 and 0.05 ppm, and corresponding calibration curves were then generated accordingly and linear interpretation was used to calculate the relative ion concentrations (ppm) from each analyte.

Elemental analysis was carried out using a Varian 710 Inductive Coupled Plasma-Optical Emission (ICP-OES) axial spectrometer (Varian, Germany) fitted with a Seaspray nebuliser. ICP-OES parameters for power, plasma flow, auxiliary flow and nebuliser pressure were 1.2 kW, 15 L/min, 1.5 L/min and 180 kPa, respectively. For optimal detection and best method for full quantification of copper, silver and zinc, three different wavelengths were selected for each element, copper 213.598, copper 324.754, copper 327.395, silver 241.318, silver 328.068, silver 338.289, zinc 202.548, zinc 206.200 and zinc 213.857. Limits of detection (LOD) and quantification (LOQ) were calculated for each wavelength by analysis of a 2% HNO_3_ blank. Elemental concentrations in all analytes were calculated using a calibration curve and weighted regression.

### Antiviral activity

*Escherichia coli* bacteriophage MS2 (ATCC 15597-B1) (used to model RNA viruses) and *Escherichia coli* bacteriophage T4 (ATCC 11303-TB4) (used to model DNA viruses) were the model organisms used throughout these experiments. Freeze-dried cultures of all strains were sourced from LGC Standards (Teddington, UK) and cultured following the manufacturers’ instructions. Stock cultures of *E. coli* ATCC 15597 and *E. coli* ATCC 11303 were stored in a Microbank™ at − 20 °C. Both bacteriophages MS2 and T4 were kept at 2 °C. Antiviral activity was assessed against these chosen microorganisms as these strains are commonly available and safe to work with in Biosafety Level 2 laboratories. Tryptone, yeast extract, sodium chloride, agar, glucose, calcium chloride, thiamine, nutrient agar and phosphate buffer saline (PBS) were purchased from Sigma-Aldrich (Gillingham, UK).

Actively growing broth cultures of *E. coli* 15597 and *E. coli* 11303 were prepared by incubating a single colony in 30 mL of sterile ATCC Medium 271 and ATCC Medium 129 broth, respectively, for 18 h at 37 °C and 150 rpm.

Bacteriophage suspensions containing 0.05, 0.1, 0.25, 0.5, 1.0 and 2.0 w/v% of copper-silver or copper-zinc nanoparticles in PBS were prepared.  100 µL of these suspensions at 0, 3 and 24 h was added to 300 µL of their respective *E. coli* and 3 mL of molten semi-solid agar (0.5% agar) and poured onto agar plates. The plates were incubated for 24 h at 37 °C and the number of plaques were counted. Viral reduction was calculated by comparing the number of virions at 3 and 24 h to 0 h.

### Antiviral nanocomposite fibres

Nanocomposite fibres were prepared from poly(methyl methacrylate) (PMMA) (Mw 120,000 g/mol) using pressurised gyration [26–30]. PMMA was chosen as a carrier material due to its favourable mechanical properties, wide range of applications, easy handling, low cost, hydrophobicity and its ability to form fibres when processed using pressurised gyration [27–30]. Copper-silver and copper-zinc nanoparticles were incorporated into the fibres at 2 wt%. The antiviral properties of the prepared fibres were assessed against bacteriophage T4. 0.1 g of fibres was incubated in 10 mL of T4 suspension for 3 h. The number of virions present at 3 h was counted using a plaque assay and compared to the positive control (no treatment) to calculate average viral reduction. A one-way ANOVA was performed to compare treatments.

## Results

### Morphological and chemical characterisation of intermetallic copper-silver and copper-zinc

**Fig. 1:**
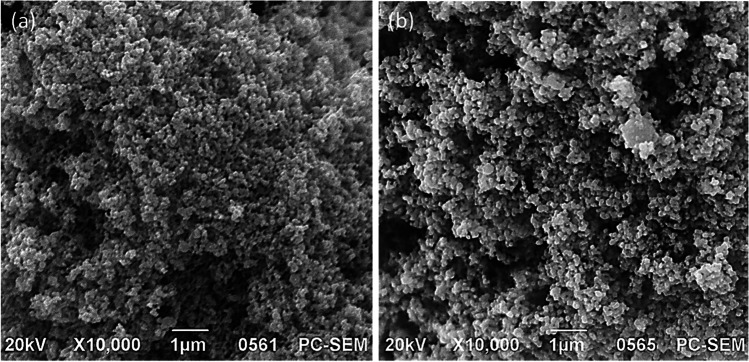
Scanning electron micrographs of (**a**) copper-silver and (**b**) copper-zinc nanoparticles

The morphology and estimated powder sizes of the two intermetallic nanoparticles were characterised by SEM. As shown in Fig. 1, the representative SEM images for intermetallic (a) copper-silver and (b) copper-zinc nanoparticles show spherical shape and appeared to be agglomerated. SEM images suggested average particle size of virgin copper-zinc  was 100–120 nm and copper-silver was 90–95 nm.

**Fig. 2:**
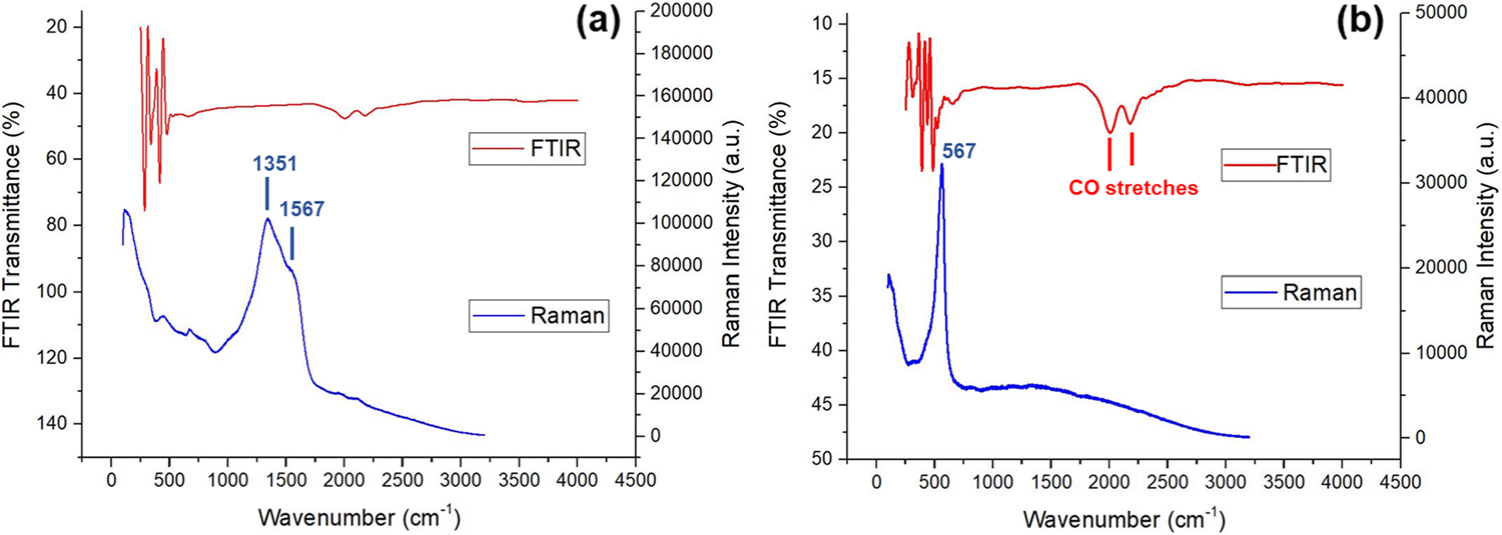
FTIR and Raman spectra of (**a**) copper-silver and (**b**) copper-zinc nanoparticles

Figure 2 shows both FTIR (red) and Raman (blue) spectra of intermetallic coppersilver (Fig. 2a) and copper-zinc  (Fig. 2b). Absorption peaks and frequencies have been identified and highlighted in Fig. 2. Both intermetallic compounds appeared to be spectroscopically Raman active. Two overlapping broad Raman bands (1351 and 1567 cm^−1^) were observed in copper-silver, whilst a single Raman shift was present at 567.8 cm^−1^ in copper-zinc.

Table 1 summarises the detectable traces of metal ions and concentrations of major ions (copper, silver and zinc) measured from fully digested copper silver  and copper-zinc  samples. Qualitative analyses performed using ICP-OES suggested both copper-silver  and copper-zinc copper-zinc contained traces of other heavy metals, such as tungsten (W), iron (Fe) and manganese (Mn). Although contaminants were detected, these metal traces were only 0.3–0.5% (w/w) of total content.

**Table 1:** ICP-OES metal trace and elemental analysis of intermetallic copper-silver (CuAg) and copper-zinc (CuZn)

**ICP digested sample**	CuAg		CuZn	
Traced ion	Ca, Mn, Zn, Br		Fe, Ni, W, Br	
Main ion	Cu	Ag	Cu	Zn
Mean (ppm)	0.08035	5.752	4.1860	2.2561
wt%	1.38	98.62	65.0	35.0
Mole ratio	0.022	0.914	1.023	0.535
Calculated formula	Cu_0.60_Ag_25_		Cu_21_Zn_11_	
Standardised formula	CuAg_42_		Cu_2_Zn	

### Antiviral activity

The viricidal properties of copper-silver  and copper-zinc  nanoparticles were tested against bacteriophages MS2 and T4. A plaque assay was used to quantify the number of infectious viral particles in suspension before and after treatment. The advantage of using plaque assays is their ability to give a direct quantitative measurement of the exact number of virions in suspension [31, 32].

**Fig. 3:**
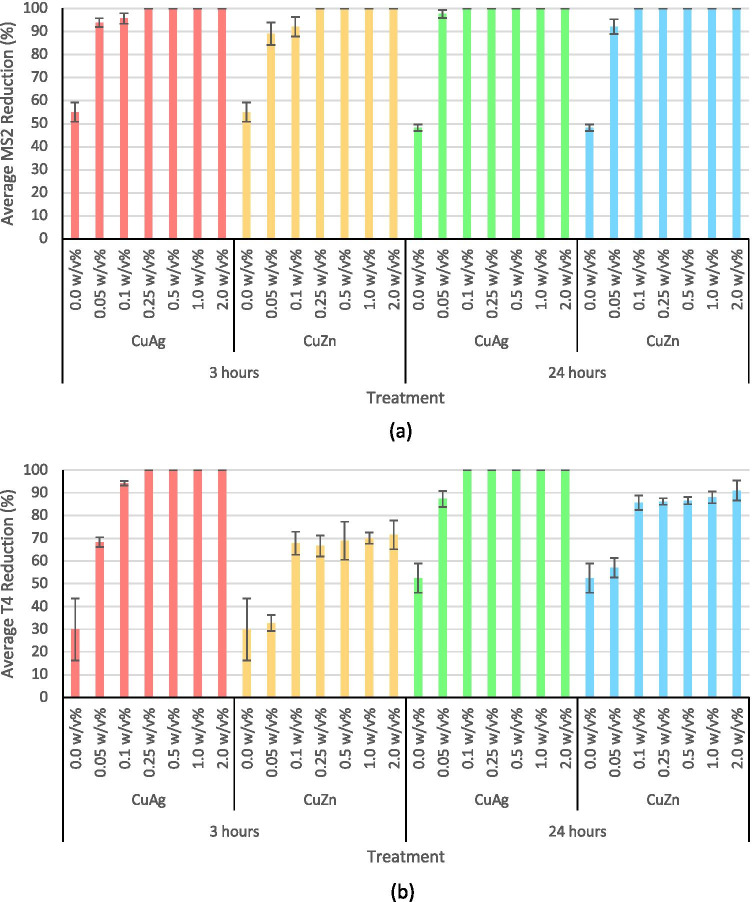
Antiviral activity of copper-silver  and copper-zinc  intermetallic nanoparticles at 0.05, 0.1, 0.25, 0.5, 1.0 and 2.0 w/v% against (a) bacteriophage MS2 for 3 and 24 h and (b) bacteriophage T4 for 3 and 24 h. Error bars represent standard deviation. 0.0 w/v% is the negative control

As seen in Fig. 3, both nanoparticles showed antiviral activity across all concentrations tested. The nanoparticles were more effective against bacteriophage MS2, when compared to bacteriophage T4. The only minor difference in antiviral activity was between 3 and 24h incubation time, thus indicating a contact time of 3 h or less is sufficient for the inactivation of MS2. At a concentration of 0.05 wt/v%, copper-zinc  intermetallic nanoparticles had a viral reduction of 89.1 ± 4.9% whilst copper-silver  nanoparticles had a viral reduction of 93.7 ± 1.9%. At 0.25 wt/v%, both nanoparticles led to complete viral inactivation.

Copper-silver  intermetallic nanoparticles were found to be more potent towards bacteriophage T4 than copper-zinc  nanoparticles. After exposure to nanoparticle treatments at 0.05 w/v% for 3 h, copper-silver  nanoparticles resulted in a reduction of 68.3 ± 2.1%, whilst for copper-zinc, it was 32.7 ± 3.5%. For a 3 h exposure time, the antiviral activity of copper-zinc  nanoparticles plateaued at 0.1 wt/v% with a reduction of 67.9 ± 5.1%, whilst copper-silver  plateaued at 0.25 wt/v% with a reduction of 100%.

**Fig. 4:**
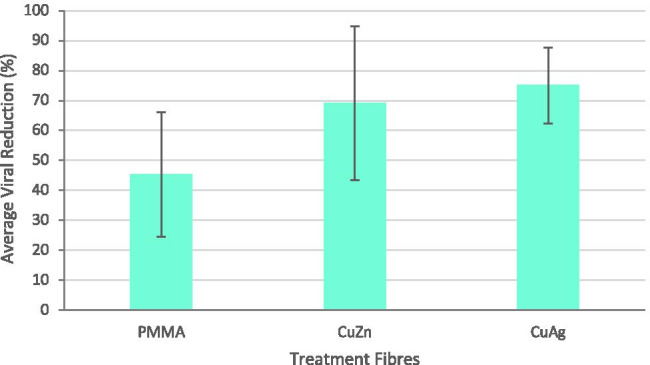
Antiviral activity of intermetallic nanocomposite fibres after 3 h incubation. Error bars represent standard deviation. Negative control represents pure PMMA fibres.

It is prudent to develop a polymeric carrier for the nanoparticles so that it can be used as antimicrobial meshes for applications like filtration. Therefore, antiviral properties of nanocomposite polymeric fibres were also assessed against bacteriophage T4 as this virus was harder to completely eradicate (Fig. 4). Viral reductions of pure polymer fibres, copper-zinc  polymer fibres and copper-silver polymer results were 45.3 ± 20.8%, 69.1 ± 25.7% and 75 ± 12.7%, respectively.

## Discussion

### Nanoparticle characterisation

The nanoparticles investigated in this study were characterised using a variety of techniques to gather information on their size, shape, morphological and chemical parameters. SEM analysis revealed the copper-zinc  intermetallic nanoparticles were slightly larger than the copper-silver  nanoparticles and a more monodispersed morphological sample was found in the  copper-silver  nanoparticles.

Typically, during FTIR analysis, the presence of metallic bonds and heavy atoms in metallic alloy materials requires a higher frequency source to induce any intra-molecular vibration/absorption beyond or near infrared region (14,000 cm^−1^). However, the lack of IR absorption signal observed in both samples also suggested that copper-silver  and copper-zinc  were free from organic contaminant, additive and moisture (ca. strong broad signal ~ 3400 cm^−1^) [33]. It is worth noting the two FTIR bands observed at 2050 cm^−1^ and 2250 cm^−1^ in both spectra (Fig. 2), vibration absorption within this range is usually associated to carbon monoxide stretch (CO), which would normally be eliminated from background scanning prior to acquisition of analyte. In our case, this could be associated to the CO chemisorbs on the open metal site at the axial position of the copper paddlewheel (a metal cluster) that is the building unit of both metal–organic frameworks (MOFs) [34].

With regard to the Raman spectra, it is believed that broad Raman bands observed in the copper-silver  nanopowder were due to the amorphous nature of the sample. Such broadening can be explained by the lack of spatial arrangement presence in their solid-state lattice. Thus, the distribution of formula units with varying bond angles and lengths produces a distribution of states of slightly varying vibrational energies, consequently resulting in signal overlapping and the observed broadening effect [35].

As the number of Raman vibrational modes depends upon the space group symmetry of the crystal, a narrow shift observed in the copper-zinc implies this sample has a consistent spatial order and crystal lattice of repeating unit cells. We were unable to find any reference to help the interpretation of this Raman shifts observed at 567.8 cm^−1^, probably because we are the first to report a Raman study of intermetallic copper-zinc  nanoparticles. The observed propagation of vibrational waves collected from copper zincc may be explained by the presence of a long-range translational symmetry associated to the copper 63.5 and zinc 65.4 intermetallic lattice in a crystalline sample.

ICP-OES was used to trace metal contaminants and to quantify major metal components present in the copper-silver and copper-zincc nanoparticles. The major metal components (copper, silver and zinc) in both copper-silver and copper-zinc were fully quantified using the emission intensity values obtained from each analysis; the corresponding weight % and molecular ratio were then subsequently calculated using external calibrated references.

As seen in Table 1, the weight ratio (wt%) of copper and silver ions found in intermetallic copper-silver was 1.38:98.62, and after incorporating their corresponding atomic mass, the final chemical formula obtained was Cu_0.60_Ag_25_. The high silver ratio implies every single intermetallic copper-silver crystal lattice contains approximately one copper atom and forty-two silver atoms. Likewise, the wt% ratio of copper and zinc (65:35) measured from ICP-OES gave the calculated formula as Cu_21_Zn_11_; hence, the standardised formula Cu_2_Zn implies every weighted quantity of the intermetallic copper-zinc nanoparticles would have twice as much of copper than zinc.

Considering the molecular weight of CuAg_42_ is almost 24 times more than Cu_2_Zn, the minimum molar concentrations of copper-silver used in the antiviral experiments (Fig. 3) gave rise to outstanding antiviral efficacies when compared to copper-zinc.

### Antiviral activity

#### Nanoparticles

The antiviral nanoparticle treatments were found to be more effective against RNA viruses than that of DNA viruses. This is thought to be due to the difference in viral structures. DNA viruses usually contain double-stranded DNA whilst RNA viruses contain single-stranded RNA [36]. As a result, DNA viruses are more robust, whilst RNA viruses are considered unstable. However, further tests are needed to approve or confirm the difference of DNA and RNA susceptibility towards these antiviral nanoparticles. It should be noted that in general, though DNA viruses are more resistant, RNA viruses are known to have high mutation rates and the ability to rapidly replace their protein coat [37, 38].

The antiviral mechanism of these nanoparticles is thought to involve copper, zinc and silver modes of their synergistic actions. The difference in potency between copper-silver  and copper-zinc intermetallic nanoparticles is attributed to the presence of silver or zinc in the compounds. The viricidal effects of the intermetallic nanoparticles are presumed to depend on the nanoparticles’ interaction with viral envelope glycoproteins, thereby inhibiting viral diffusion and penetration into the host cell [39]. In a study by Baram-Pinto et al. [40], silver metallic nanoparticles were shown to target and bind to viruses, such as herpes simplex virus, thus preventing its entry into the host cell and subsequent infection. Huy et al. [41] also reported a similar mechanism whereby silver metallic nanoparticles bind with viral particles, thus preventing the virus from entering the host cell and replicating the number of extracellular virions. Fujimori et al. [42] also demonstrated copper nanoparticle-induced degradation of viral glycoproteins (such as hemagglutinin) thus preventing the virus from binding to the host cells. Researchers have also reported the inhibitory effect of zinc nanoparticles is thought to be the result of zinc nanoparticles attaching to virion surfaces thus consequently preventing the virus from binding to and penetrating the host cell [43–45]. Therefore, the mechanistic understanding of the antiviral action of these intermetallic nanoparticles is thought to involve the nanoparticles interacting with the viral surface proteins and prevent them from entering the host cell.

When compared to existing literature, intermetallic copper-silver nanoparticles had stronger antiviral properties than single elemental nanoparticles. Fujimori et al. [42] reported a 50% reduction when influenza A (RNA virus) was exposed to 17 g/mL of copper iodide nanoparticles, whilst other studies have reported a 50% reduction when hepatitis B (DNA virus) and human immunodeficiency virus-1 (RNA virus) were exposed to silver nanoparticles [40, 42]. In this study, a maximum nanoparticle concentration of 0.2 g/mL was used, thereby suggesting the use of intermetallic alloys is favourable when compared to single elemental metallic nanoparticles.

Copper-zinc intermetallic nanoparticles exhibited synergistic antiviral properties across all three concentrations tested, with a maximum viral reduction of 96.9 ± 0.3% being achieved at 2 wt/v%. These nanoparticles were not as potent as the copper-silver nanoparticles; this observation can be attributed to the presence of zinc in replacement of silver. Tavakoli et al. [46] reported zinc oxide nanoparticles resulted in a 33% antiviral inhibition rate when used at a concentration of 100 µg/mL against herpes simplex virus type 1 (DNA virus). When compared to existing literature, intermetallic copper-zinc nanoparticles have stronger antiviral properties than single elemental nanoparticles.

#### Nanocomposite fibres

Combining the antiviral agents with carrier polymeric matrices improves the materials usability, allowing it to be effectively used in industrial, commercial and consumer applications. Therefore, copper-silver and copper-zinc intermetallic nanoparticles were incorporated into polymeric fibres as previously described [26–29], to determine whether or not the nanoparticles remain effective against viruses. As shown in Fig. 4, pure polymer (PMMA) fibres showed a moderate decrease in virions. This reduction can be attributed to two factors: (i) the lack of host cells in the PBS to allow for viral survival and reproduction; (ii) viral damage (and possible viral binding to the PMMA fibre surfaces) caused by the hydrophobic interaction between the hydrophobic surface of PMMA and the hydrophobic proteins present on the virus surface [47–49].

Fibres containing copper-silver nanoparticles exhibited slightly stronger antiviral properties than fibres containing copper-zinc intermetallic nanoparticles. These results are in agreement with the observations made regarding the nanoparticles. Statistical analysis (one-way ANOVA) showed no significant difference between the copper-zinc intermetallic nanoparticles and the copper-zinc fibres (*F*-statistic = 0.01755, *P*-value = 0.90099) indicating the nanoparticles and fibres performed equally well, whilst copper-silver intermetallic nanoparticles and copper-silver fibres showed a significant difference (*F*-statistic = 13.02083, *P*-value = 0.02259) suggesting the copper-silver nanoparticles lost efficacy or efficiency when incorporated into the fibres.

The toxic effect of intermetallic nanoparticles on viruses is evident from this research; however, their effect on human cells needs to be further investigated. Existing literature gives conflicting opinions and some articles state metallic nanoparticles are cytotoxic [50–62], whilst others state the composited nanoparticles are not cytotoxic to mammalian cells and can be used in various biomedical constructs and applications [63–69].

## Conclusions

This research showcases, for the first time, the antiviral properties of intermetallic copper-silver and copper-zinc nanoparticles. Understanding the antiviral properties of these nanoparticles and their synergistic activities is critical for their future application. Antiviral studies revealed both nanoparticles are potent towards RNA viruses, whilst copper-silver nanoparticles were more effective towards DNA viruses. These materials present themselves as suitable alternatives in a wide range of antimicrobial applications. Their ability to remain potent when incorporated into polymeric fibres demonstrates the potential of these nanoparticles in a range of applications, spanning from environmental to biomedical engineering. The exploitation of these functionalised materials is a viable solution to help alleviate some of the burden on healthcare services caused by viruses. The outcome of this research provides the necessary guidance for the utilisation of antiviral nanoparticles to mitigate the transmission of viruses. The knowledge presented here can be applied to the healthcare sector, as well as the hospitality sector, retail, transportation and much more.

## Data Availability

All data is provided in the manuscript.
